# Factors associated with adherence to colonoscopy among individuals who were positive in the preliminary screening for colorectal neoplasms

**DOI:** 10.1002/cam4.4730

**Published:** 2022-04-20

**Authors:** Ji‐Bin Li, Keng‐Jian Ke, Wei‐Li Zhang, Ling‐Yan Wang, Yan‐Ping Wu, Fan Weng, Huan Tian, Zhi‐Yu Qiu, Yin Li, Shi‐Yong Lin, Mei‐Xian Ye, Qing‐Jian Ou, Cheng‐Hua Gong, Zhen‐Hai Lu, Zhi‐Zhong Pan, De‐Sen Wan, Jian‐Hong Peng, Yu‐Jing Fang

**Affiliations:** ^1^ Sun Yat‐sen University Cancer Center State Key Laboratory of Oncology in South China, Collaborative Innovation Center for Cancer Medicine Guangzhou Guangzhou P. R. China; ^2^ Shenzhen Hospital Southern Medical University Shenzhen P. R. China; ^3^ Yuexiu District Center for Disease Control and Prevention Guangzhou P. R. China; ^4^ Dadong Street Community Health Service Center Guangzhou P. R. China

**Keywords:** adherence to colonoscopy, colorectal cancer screening, fecal immunochemical test, high‐risk factor questionnaire

## Abstract

**Objectives:**

This study aimed to investigate the potential factors associated with adherence to colonoscopy among participants who were preliminarily screened positive in a community‐based colorectal cancer screening program in China.

**Methods:**

This study analyzed data from 1219 out of 6971 community residents who were identified as positive cases by the well‐validated high‐risk factor questionnaire (HRFQ) or fecal immunochemical test (FIT) in the preliminary screening stage for colorectal neoplasms. Patients showing adherence to colonoscopy were defined as those who received positive results in a preliminary screening for colorectal neoplasms and later received a colonoscopy examination as required. The associations of social‐demographic factors, lifestyle behaviors, history of diabetes, body mass index (BMI), and risk factors in the HRFQ with adherence to colonoscopy were evaluated using logistic regression models.

**Results:**

Among 1219 participants who preliminarily screened positive, the top five risk factors reported by the participants were chronic constipation (25.9%), hematochezia (23.5%), family history of CRC in first‐degree relatives (22.1%), chronic diarrhea (21.8%), and history of polyps (16.6%). Around 14.2% of participants who preliminarily screened positive reported three or more risk factors, and the proportion was 26.2% among participants who were positive according to both HRFQ and FIT. Among all participants who were preliminarily screened positive, the multivariable results showed that those who were married (OR = 1.58, 95% CI: 1.12, 2.25, *p* = 0.01), had chronic diarrhea (OR = 1.34, 95% CI: 1.00, 1.78, *p* = 0.047), and had a positive FIT (OR = 1.60, 95% CI: 1.21, 2.10, *p* < 0.001 for patients who were negative according to HRFQ but positive according to FIT; OR = 2.12, 95% CI: 1.33, 2.78, *p* = 0.002 for patients who were positive for both HRFQ and FIT) were more likely to adhere to colonoscopy, while participants with a history of cancer (OR: 0.50, 95% CI: 0.31, 0.79, *p* = 0.003) were less likely to adhere to colonoscopy. The results among participants who were tested positive according to only HRFQ were similar to those among all participants who were tested positive according to HRFQ or FIT. However, among participants who were tested positive according to only FIT, we only found that those who were married (OR = 2.52, 95% CI: 1.08, 5.90, *p* = 0.033) had a higher odds of adhering to colonoscopy, while those with a history of diabetes (OR = 0.35, 95% CI: 0.13, 0.96, *p* = 0.042) were less likely to adhere to colonoscopy.

**Conclusion:**

Our findings provide evidence supporting the development of tailored interventional strategies that aim to improve adherence to colonoscopy for individuals with a high risk of colorectal neoplasms. Both barriers and facilitators associated with adherence to colonoscopy should be considered in supportive systems and health policies. However, further well‐designed prospective studies are warranted to confirm our findings.

## Graphical abstract

For the participation rate of colonoscopy screening is still disappointingly low in China. We find out the factors associated with colonoscopy adherence among individuals who were positive in the preliminary screening for colorectal neoplasms.

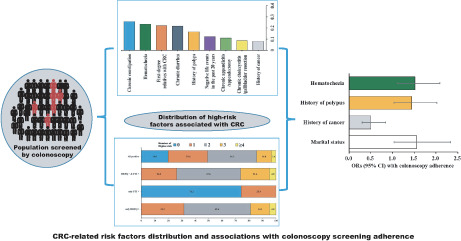

## 1 INTRODUCTION

The International Agency for Research on Cancer reported that colorectal cancer (CRC) was the second leading cause of cancer death worldwide in 2020.^1^ The global burden of CRC is estimated to increase by 60% to more than 2.2 million new cases and 1.1 million deaths by 2030.^2^ In China, an increasing trend of CRC incidence was observed and will continue to increase in future decades due to the rise of the aging population.^3^ The incidence and mortality of CRC ranked third (age‐standardized rate: 18.02/100,000) and fifth (age‐standardized rate: 8.21/100,000) out of all malignant tumors in China in 2015.^4^

Cumulative evidence proves that early screening and detection of colorectal neoplasms could significantly reduce CRC‐specific incidence and mortality in the general population.^5^, ^6^ For instance, fecal immunochemical test (FIT) screening could reduce CRC incidence by 10%^7^ and CRC mortality by 62%.^8^ Colonoscopy, as the gold standard for detecting colorectal neoplasia, was associated with a 68% reduction in CRC‐specific mortality compared with no colonoscopy.^9^ Throughout the world, there are widespread differences in the implementation status and strategies for CRC screening.^5^, ^10^ Colonoscopy is applied as a primary screening tool in some developed countries.^11^ In China, a two‐step sequential screening strategy is officially recommended^12^: eligible individuals are preliminarily screened by a well‐validated high‐risk factor questionnaire (HRFQ) or FIT, and cases identified as positive in the preliminary screening are further referred to receive confirmation from a colonoscopy.^13^

Apart from the good performance of the CRC screening test, its effectiveness and early diagnostic rate are partially determined by the rate of adherence to colonoscopy among the population that is positive according to the preliminary screening. However, low adherence to colonoscopy has been reported in different regions of China.^14^ For instance, in mass screening in Guangzhou from 2015 to 2017, a 19.8% rate of adherence to colonoscopy was reported,^15^ and the rate was 39.8% in Shanghai in 2013.^16^ The Chinese national urban cancer screening program in 14 cities reported that only 33.3% of residents positive at the preliminary screening underwent colonoscopy.^17^ In a recent randomized controlled trial aiming to improve the rate of adherence to colonoscopy among the population that was positive at a preliminary screening in Guangzhou, it was reported that the rates of adherence to colonoscopy were only 7.1%, 9.6%, and 13.7% at the sixth month in the control, low‐frequency intervention, and high‐frequency intervention groups, respectively.^18^

The rate of participation in colonoscopy screening is still disappointingly low in China. It is therefore important to understand the factors that are associated with adherence to colonoscopy among the population who are positive at the preliminary screening, which would be useful in order to design tailored interventional programs to improve the effectiveness and early detection rate of CRC screening. A few studies based on the health belief model have explored the factors associated with adherence to colonoscopy, reporting that higher levels of signals for action, lower perceived knowledge barriers, and severity and fear were significantly associated with higher odds of adhering to CRC screening.^19^, ^20^ In the present study, using data from a large‐scale population‐based CRC screening program in Guangzhou, we systematically investigated the potential factors associated with adherence to colonoscopy among preliminary screening‐positive participants, including social‐demographic factors, lifestyle behaviors, history of diabetes, body mass index (BMI), and risk factors in the HRFQ.

## 2 METHODS

### 2.1 Screening strategy and participants

**FIGURE 1:**
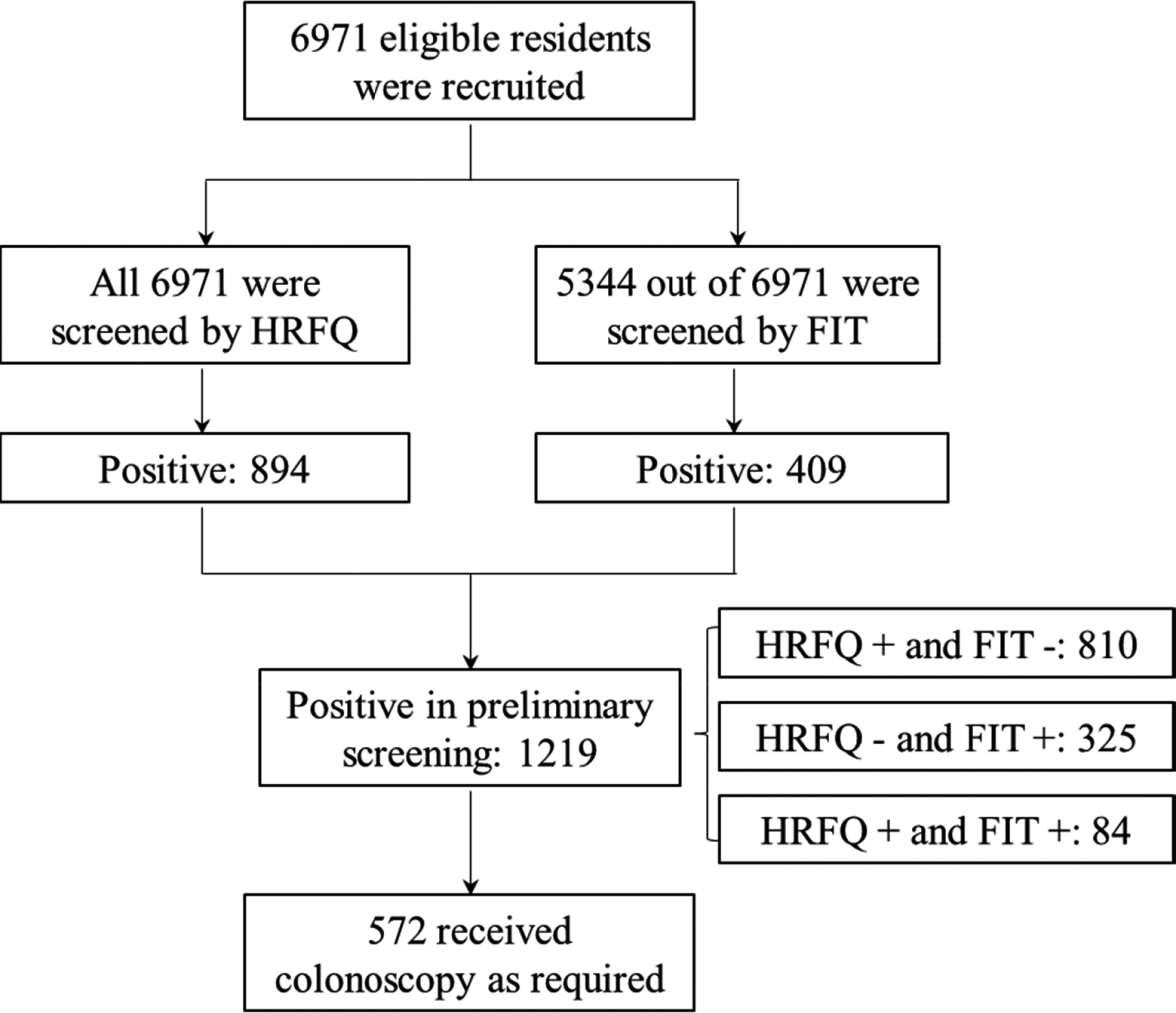
Flowchart of participants. FIT, fecal immunological test; HRFQ, high‐risk factor questionnaire

A population‐based screening program for colorectal neoplasms in Guangzhou, China, was launched in 2014.^21^ The eligible participants in the screening program were community residents aged 50–74 years. A total of 6971 participants were preliminarily screened by HRFQ or FIT at the end of December 2018. In this study, those with positive results in the preliminary screening stage were involved in the analysis (Figure 1).

A two‐step screening strategy was applied based on the recommendation by the National Health Commission of the People's Republic of China. The community residents aged 50–74 years were preliminarily screened by HRFQ or FIT, and those with positive results according to HRFQ or FIT were identified as being at high risk for CRC and were further referred to colonoscopy confirmation. Individuals were defined as being at high risk for CRC by HRFQ if they had a personal history of cancers, history of CRC in first‐degree relatives, or history of polyps or had at least two of the following symptoms: chronic constipation or diarrhea, hematochezia, history of appendicitis, history of cholecystitis, or history of psychiatric trauma (e.g., divorce, death of relatives) in the past 20 years. The FIT was applied to detect occult blood in stools. All participants were provided with two collection kits (supplied by ABON, China) and required to collect 10–50 mg of stool twice in two consecutive weeks according to the manufacturer's recommendations. Stool samples were sent to local community health centers within 6 h of collection. All participants were required to undergo a second test regardless of the result of the first FIT. Among 6971 participants, 76.7% completed the first FIT, and 65.5% completed two FITs. Participants with positive results from the preliminary screening stage were referred to Sun Yat‐sen University Cancer Center or other authorized medical centers for colonoscopy confirmation.

### 2.2 Potential predictors

Participants' social‐demographic characteristics of age, gender, height in meters, weight in kilograms, marital status, education level, and history of diabetes were self‐reported. Lifestyle behaviors of smoking (categorized as never, formerly, or currently smoking), alcohol drinking (categorized as no or yes), history of night work (categorized as no or yes), and proportion of sedentary time in a typical working day (categorized as <50% and ≥50%) were collected by a self‐administrated questionnaire. Body mass index (BMI) was calculated as weight (kg) divided by height squared (m). Overweight or obesity was defined as BMI ≥25 kg/m^2^ based on the World Health Organization guideline.

### 2.3 Statistical analysis

Participants with the outcome of adherence to colonoscopy were defined as those who had positive results in the preliminary screening and further received colonoscopy as required by the protocol.

Social‐demographic variables, lifestyle‐related factors, and high‐risk factors in HRFQ were described using frequency with a percentage, stratified by preliminary screening results. Univariable odds ratios along with 95% confidence intervals (95% CIs) of potential factors associated with adherence to colonoscopy were initially derived. Predictors associated with adherence to colonoscopy in univariable analyses at *p* < 0.10 level were further included in multivariable logistic regression models for risk factor selection using the stepwise method. Sensitivity analyses were then conducted among participants who were positive for CRC according to only HRFQ or only FIT.

All analyses were performed using SAS software version 9.4 (SAS Institute Inc.). A *p*‐value was based on two‐sided tests and considered statistically significant at *p* < 0.05.

### 2.4 Ethics

This study was approved by the institutional review board of Sun Yat‐sen University Cancer Center, and written informed consent was obtained from all participants. The authenticity of this article has been validated by uploading the key raw data onto the Research Data Deposit platform (www.researchdata.org.cn) with the approval RDD number of RDDA2019001156.

## 3 RESULTS

### 3.1 Participants' characteristics

Table 1 shows participants' characteristics stratified by preliminary screening results. All 6971 participants were preliminarily screened by HRFQ, and 5344 out of 6971 were screened by at least one FIT. A total of 1219 participants (17.5%) were identified as positive cases in the preliminary screening stage, including 810 (66.5%) with only positive HRFQ results, 325 (26.7%) with only positive FIT results, and 84 (6.9%) with both positive HRFQ and positive FIT results. A total of 572 out of 1219 preliminarily positive participants (46.9%) further received a colonoscopy (Figure 1).

**TABLE 1:** Sample characteristics stratified by preliminary screening results

	Total	Preliminary screening results			
		Only HRFQ+	Only FIT+	HRFQ+ and FIT+	All positive
All	6971	810	325	84	1219
Gender, *n* (%)					
Female	4211 (60.4)	507 (62.6)	188 (57.9)	44 (52.4)	739 (60.6)
Male	2760 (39.6)	303 (37.4)	137 (42.1)	40 (47.6)	480 (39.4)
Age, mean ± SD, *n* (%)	60.0 ± 7.2	59.4 ± 7.6	61.2 ± 7.7	60.1 ± 8.1	59.9 ± 7.7
≤60 years	3725 (53.4)	450 (55.6)	142 (43.7)	35 (41.7)	627 (51.4)
>60 years	3246 (46.6)	360 (44.4)	183 (56.3)	49 (58.3)	592 (48.6)
Marital status, *n* (%)					
Single/divorce/bereft spouse	674 (9.7)	127 (15.7)	26 (8.0)	13 (15.5)	166 (13.6)
Married	6297 (90.3)	683 (84.3)	299 (92.0)	71 (84.5)	1053 (86.4)
Education level, *n* (%)					
Primary school or below	635 (9.1)	74 (9.1)	30 (9.2)	4 (4.8)	108 (8.9)
Middle school	5169 (74.2)	509 (62.8)	241 (74.2)	51 (60.7)	801 (65.7)
College or above	1167 (16.7)	227 (28.0)	54 (16.6)	29 (34.5)	310 (25.4)
Former/current smoker, *n* (%)	319 (4.6)	62 (7.7)	21 (6.5)	9 (10.7)	92 (7.5)
Alcohol drinking, *n* (%)	97 (1.4)	25 (3.1)	4 (1.2)	2 (2.4)	31 (2.5)
History of night work, *n* (%)	283 (4.1)	65 (8.0)	19 (5.9)	6 (7.1)	90 (7.4)
Sedentary for more than half of working time, *n* (%)	1306 (18.7)	218 (26.9)	93 (28.6)	33 (39.3)	344 (28.2)
History of diabetes, *n* (%)	282 (4.1)	46 (5.7)	19 (5.8)	5 (6.0)	70 (5.7)
Overweight or obesity, *n* (%)	610 (8.7)	110 (13.6)	44 (13.5)	12 (14.3)	166 (13.6)

Abbreviations: FIT, fecal immunological test; HRFQ, high risk factor questionnaire; SD, standard deviation.

Among 1219 participants who were tested positive in the preliminary screening stage, the mean age was 60.0 years (standard deviation: 7.2), 60.6% were females, 25.4% received an education of college or higher, and the majority of participants (86.4%) were married. The proportions of overweight or obesity, formerly/currently smoking, alcohol drinking, history of diabetes, and history of night work were 13.6%, 7.5%, 2.5%, 5.7%, and 7.4%, respectively. In addition, around 28.2% of positive cases reported more than 50% of time spent sedentarily in their typical working days (Table 1).

### 3.2 Distribution of risk factors by preliminary screening results

**FIGURE 2:**
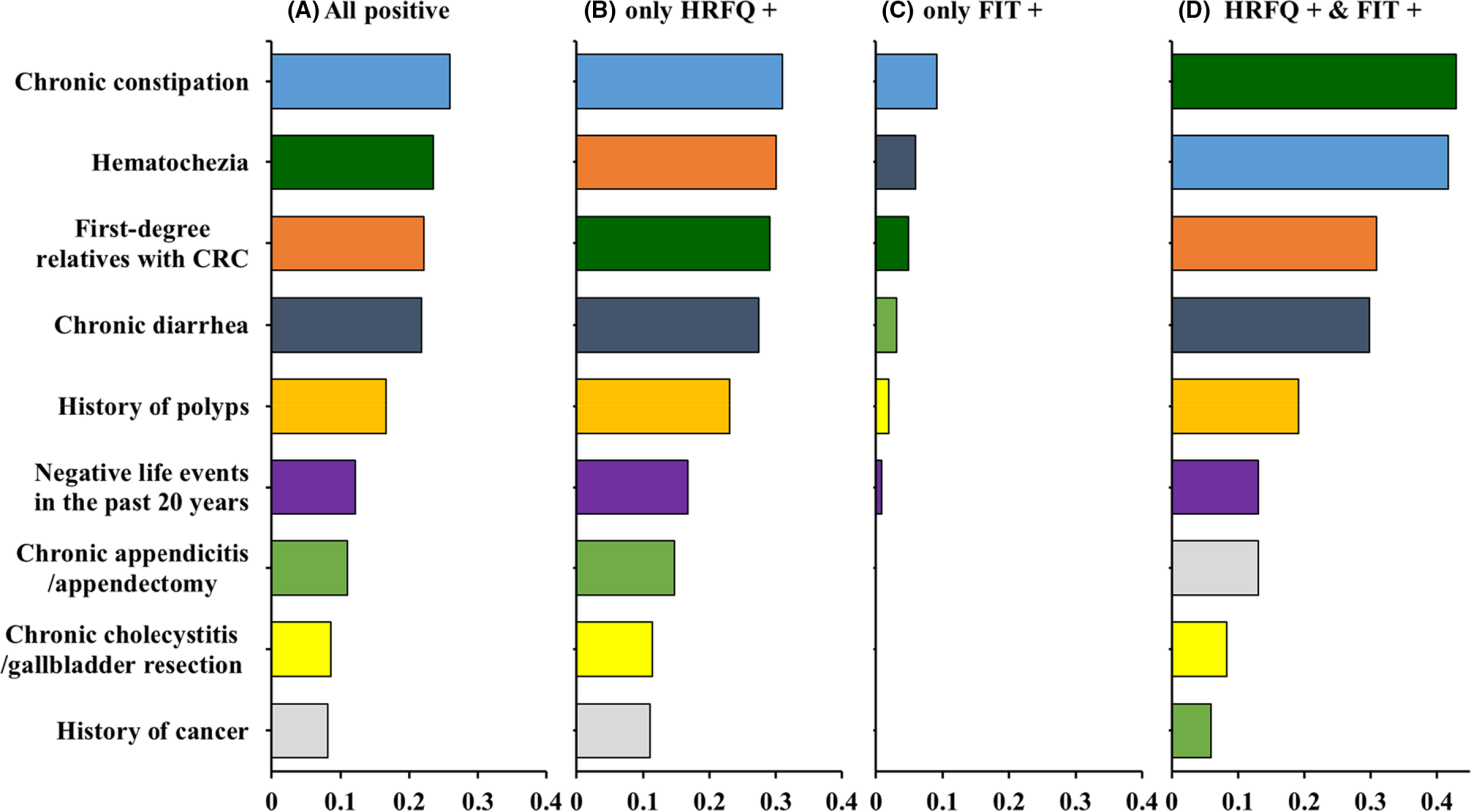
Distribution of risk factors by preliminary screening results. The same color represents the same risk factor; e.g., blue represents chronic constipation. CRC, colorectal cancer; FIT, fecal immunological test; HRFQ, high‐risk factor questionnaire

**FIGURE 3:**
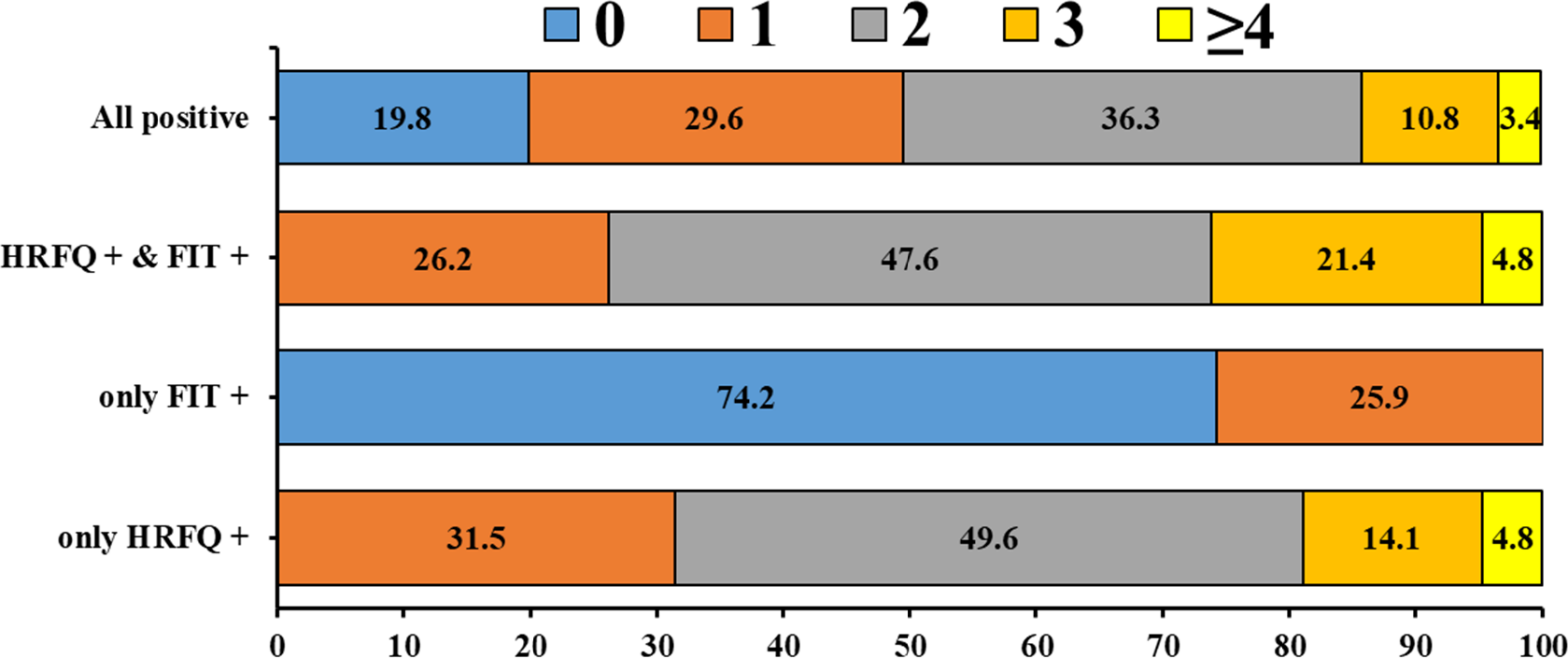
Distributions of number of risk factors stratified by preliminary screening results. FIT, fecal immunological test; HRFQ, high‐risk factor questionnaire

In all nine risk factors of HRFQ, the top five risk factors reported by participants were chronic constipation (25.9%), hematochezia (23.5%), family history of CRC in first‐degree relatives (22.1%), chronic diarrhea (21.8%), and history of (16.6%) among all positive participants (Figure 2). The top five risk factors among cases that were positive according to only HRFQ and cases that were positive according to both HRFQ and FIT were the same as those among all positive participants but were ranked differently (Figure 2). Around one‐seventh (14.2%) of all positive participants reported three or more risk factors, and the proportion was 26.2% among participants who were positive according to both HRFQ and FIT (Figure 3).

### 3.3 Factors associated with adherence to colonoscopy

After adjusting for gender and age, the multivariable results showed that participants who were married (ORm: 1.58, 95% CI: 1.12, 2.25, *p* = 0.01), had chronic diarrhea (ORm: 1.34, 95% CI: 1.00, 1.78, *p* = 0.047), and had a positive FIT result (ORm: 1.60, 95% CI: 1.21, 2.10, *p* < 0.001 for only positive FIT; ORm: 2.12, 95% CI: 1.33, 3.78, *p* = 0.002 for both positive HRFQ and positive FIT) were more likely to adhere to colonoscopy examination. However, participants who had a history of cancer (ORm: 0.50, 95% CI: 0.31, 0.79, *p* = 0.003) were less likely to adhere to colonoscopy examination (Table 2).

**TABLE 2:** Factors associated with adherence to colonoscopy among all positive participants

	Adherence to colonoscopy, *n* (%)	Univariate		Multivariable	
		OR (95% CI)	*p*	OR (95% CI)	*p*
Gender					
Female	351 (47.5)	1		1	
Male	221 (46.0)	0.94 (0.75, 1.19)	0.619	0.88 (0.69, 1.12)	0.286
Age					
≤60 years	308 (49.1)	1		1	
>60 years	264 (44.6)	0.84 (0.67, 1.04)	0.114	0.83 (0.66, 1.05)	0.126
Marital status					
Single/divorced/bereft spouse	61 (36.8)	**1**		**1**	
Married	511 (48.5)	**1.62 (1.16, 2.28)**	**0.005**	**1.58 (1.12, 2.25)**	**0.010**
Education level					
Primary school or below	39 (36.1)	**1**		ns	
Middle school	379 (47.3)	**1.59 (1.05, 2.41)**	**0.029**		
College or above	154 (49.7)	**1.75 (1.11, 2.74)**	**0.016**		
Smoking					
Never	232 (47.6)	1		–	
Former/current smoker	47 (51.1)	1.15 (0.74, 1.79)	0.543		
Alcohol drinking					
No	263 (48.0)	1		–	
Yes	16 (51.6)	1.56 (0.56, 2.39)	0.694		
History of night work					
No	235 (49.7)	1		–	
Yes	39 (43.3)	0.77 (0.49, 1.22)	0.270		
Sedentary more than half the time at work					
No	105 (47.7)	1		–	
Yes	168 (48.8)	1.05 (0.75, 1.47)	0.797		
History of diabetes					
No	244 (49.2)	**1**		ns	
Yes	26 (37.1)	**0.65 (0.40, 1.07)**	**0.093**		
Overweight or obesity					
No	202 (50.5)	1		–	
Yes	72 (43.4)	0.75 (0.52, 1.08)	0.123		
*Risk factors*					
First‐degree relatives with CRC					
No	459 (48.3)	**1**		ns	
Yes	113 (42.0)	**0.78 (0.59, 1.02)**	**0.068**		
History of cancer					
No	543 (48.5)	**1**		**1**	
Yes	29 (29.0)	**0.43 (0.28, 0.68)**	**<0.001**	**0.50 (0.31, 0.79)**	**0.003**
History of polypus					
No	468 (46.0)	1		–	
Yes	104 (51.5)	1.25 (0.92, 1.68)	0.155		
Chronic constipation					
No	425 (47.1)	1		–	
Yes	147 (46.5)	0.98 (0.76, 1.27)	0.867		
Chronic diarrhea					
No	435 (45.7)	**1**		**1**	
Yes	137 (51.5)	**1.27 (0.96, 1.66)**	**0.091**	**1.34 (1.00, 1.78)**	**0.047**
Hematochezia					
No	424 (45.5)	**1**		ns	
Yes	148 (51.6)	**1.28 (0.98, 1.66)**	**0.072**		
Chronic appendicitis/appendectomy					
No	511 (47.1)	1		–	
Yes	61 (45.5)	0.94 (0.66, 1.35)	0.731		
Chronic cholecystitis/gallbladder resection					
No	528 (47.4)	1		–	
Yes	44 (41.9)	0.80 (0.53, 1.20)	0.282		
Negative life events					
No	504 (47.1)	1		–	
Yes	68 (45.6)	0.94 (0.67, 1.33)	0.738		
*Preliminary screening results*					
HRFQ+ and FIT−	345 (42.6)	**1**		**1**	
HRFQ− and FIT+	177 (54.5)	**1.61 (1.24, 2.09)**	**<0.001**	**1.60 (1.21, 2.10)**	**<0.001**
HRFQ+ and FIT+	50 (59.5)	**1.98 (1.26, 3.13)**	**0.003**	**2.12 (1.33, 3.78)**	**0.002**

Bold values indicated the value had statistical significance.

Abbreviations: –, not applicable; 95% CI: 95% confidence interval; BMI, body mass index; CRC, colorectal cancer; FIT, fecal immunological test; HRFQ, high‐risk factor questionnaire; ns, nonsignificant; OR, odds ratio.

We conducted sensitivity analyses in participants who were positive according to only HRFQ and were positive according to only FIT. The results among participants who were positive according to only HRFQ were similar to those among all positive participants. After adjusting for gender and age, the multivariable results showed that participants who were married (ORm: 1.55, 95% CI: 1.03, 2.33, *p* = 0.036), had a history of polyps (ORm: 1.44, 95% CI: 1.03, 2.02, *p* = 0.036), or had symptoms of hematochezia (ORm: 1.52, 95% CI: 1.11, 2.09, *p* = 0.009) were more likely to adhere to colonoscopy examination, while those with a history of cancer were less likely to adhere to colonoscopy (ORm: 0.50, 95% CI: 0.30, 0.84, *p* = 0.009) (Table S1).

Among participants who were positive according to only FIT, we only found that marital status was significantly associated with higher odds of adherence to colonoscopy (ORm: 2.52, 95% CI: 1.08, 5.90, *p* = 0.033), but a history of diabetes was significantly associated with lower odds of adherence to colonoscopy (ORm: 0.35, 95% CI: 0.13, 0.96; *p* = 0.042), after adjusting of gender and age (Table S2).

## 4 DISCUSSION

In this study, we found that high‐risk individuals identified in the preliminary screening who were married or had symptoms of chronic diarrhea, polypus, or hematochezia were most likely to adhere to colonoscopy. Specifically, HRFQ‐positive individuals with symptoms of hematochezia or a history of polyps had a higher likelihood of adhering to colonoscopy. However, positive cases with a history of chronic diseases (i.e., cancers or diabetes) were less likely to adhere to colonoscopy. These findings provide a better understanding of the potential barriers and facilitators associated with adherence to colonoscopy among populations preliminarily screened as positive and could be helpful for developing tailored interventional strategies aiming to improve colonoscopy compliance in Chinese high‐risk population.

In our study, the compliance rate of colonoscopy among preliminarily screened positive participants was 46.9%, which was higher than that reported in previous studies.^15^, ^16^ This might be partially attributed to the following reasons: First, those preliminarily screened positive participants were given priority for colonoscopy examination by being fast‐tracked in their respective hospitals/centers. Second, colonoscopy was freely provided to residents in the current screening program. Third, participants who were initially screened positive were reminded to undergo colonoscopy and were also given health education by our staff, increasing their willingness to receive colonoscopy. Fourth, the selected hospitals/centers providing colonoscopy examination were near where the community lived, which could have made available colonoscopy significantly more convenient. These factors might help improve the compliance of residents with respect to their participation in CRC screening programs.

Chronic constipation, hematochezia, family history of CRC in first‐degree relatives, chronic diarrhea, and history of polyps were the most frequently reported risk factors among preliminarily screened positive populations. Approximately one in seven (14.2%) preliminarily screened positive individuals reported three or more risk factors, which increased to 26.2% among individuals tested positive according to both HRFQ and FIT. An interesting finding was that up to 74.2% of individuals positive according to only FIT did not report any CRC‐related risk factors, while the proportion was 19.8% among all preliminarily screened positive individuals. FIT, a widely applied method for detecting hemoglobin in the stool with acceptable sensitivity, has been recommended for CRC screening worldwide.^22^ However, it is well known that FIT is less sensitive than HRFQ for nonbleeding or intermittent bleeding lesions,^12^ and this fact was confirmed by the findings in our study. The history of polyps and CRC in first‐degree relatives was completely absent in individuals who were positive according to only FIT in the preliminary screening stage. HRFQ, a well‐validated screening questionnaire,^13^, ^23^ could undoubtedly compensate for such information being missed by FIT in the preliminary screening. Consistent with previous reports,^12^, ^24^ our findings indicated that the combination of FIT and HRFQ could effectively increase the early detection rate of individuals who were at a high risk for CRC. However, our study found that some risk factors were disproportionately reported by participants, indicating that different risk factors had different contributions to identifying individuals at high risk. Therefore, mathematical quantification of these risk factors with different weights might further improve preliminary screening accuracy in future work.

Our findings indicate that married individuals were more likely to adhere to colonoscopy examination if they were identified as being at a high risk for CRC in the preliminary screening stage, which is consistent with previous reports.^25^, ^26^ One possible explanation is that married individuals have a high level of responsibility for their spouses and could therefore receive emotional and social support from their family, thus promoting healthy behaviors.^27^ As a result, married individuals are more likely to adhere to colonoscopy than unmarried individuals, even with the same health conditions.^28^ Furthermore, health concerns from a spouse could encourage colonoscopy examination.^29^ Previous studies reported that individuals usually choose spouses with similar health‐related attitudes and behaviors^30^ and that the lifestyles of married individuals are more likely to be healthy and regular.^31^, ^32^ In addition, married individuals tend to have higher insurance coverage and relatively good financial support from their families compared to unmarried individuals, which improves their adherence to colonoscopy.^29^, ^33^

The compliance with colonoscopy was significantly higher among individuals with a preliminary positive result who had a history of polyps, symptoms of hematochezia, or chronic diarrhea. These three risk factors are overt symptoms that individuals can directly perceive and that may cause personal psychological and physical discomfort.^34^ As pointed out in the health belief model, when individuals perceived that they were at a high risk for negative health outcomes, they were more likely to seek preventive behaviors (e.g., regular cancer screening, physical examination.).^35^, ^36^ These overt symptoms alert individuals' fear of disease and worries about their well‐being, increasing the likelihood of seeking out medical interventions. In addition, individuals who have regular physical examinations are more likely to contact the healthcare system, which also makes it more convenient to undergo a colonoscopy.^37^ Furthermore, the history of intestinal polyps and symptoms of hematochezia promote vigilance in seeking help from physicians for potential intestinal tumors. Long‐term chronic diarrhea also needs to be differentiated from intestinal tuberculosis, Crohn's disease, and ulcerative colitis. In these circumstances, a colonoscopy would be highly recommended by physicians for the diagnosis and the differentiation of CRC from other organic intestinal diseases.^38^

An interesting finding is that individuals with a history of chronic diseases (e.g., cancer or diabetes) had a lower rate of compliance with colonoscopy screening, which is consistent with results from South Korea and the United States.^39^, ^40^ A personal history of diabetes has a negative influence on cancer screening compliance in the elderly,^41^ including participants in our study. It is known that the elderly are a population with a high risk for comorbidity (e.g., diabetes, cancers, or obesity).^42^, ^43^ The burden of primary care for comorbidity may lead to a lower compliance rate of cancer screening, considering that utilization of preventive care was lower when the number of eligible preventive services was higher.^44^ In addition, the elderly with comorbidity (e.g., obesity or type 2 diabetes) might have unhealthy lifestyle habits and weaker health awareness than the non‐elderly,^45^ leading to lower participation in healthcare services (e.g., cancer screening).^32^ Individuals with a history of cancer might have undergone detailed serological and noninvasive imaging examinations during the regular review process. These examinations partially rule out the probability of intestinal malignancies. Due to the cost and discomfort of colonoscopy, physicians are also less likely to refer patients to colonoscopy when there is no definite evidence of suspected intestinal malignancy.^46^ Moreover, the preparation and procedure associated with colonoscopy along with other discomforts (e.g., abdominal pain after colonoscopy) would further damage the elderly's compliance with cancer screening.^20^

Our findings provide evidence that policy‐makers should develop tailored interventional strategies that aim to improve colonoscopy compliance in the Chinese population at a high risk for CRC. Health education from physicians may increase this population's awareness of the necessity and benefits of colonoscopy. However, there are several limitations to be cautious of when interpreting the results in this study. First, there might be a recall bias due to the method of self‐reported data collection for risk factors. Second, participants were conveniently sampled from community residents in the urban areas of Guangzhou, there might be selection bias. Third, as our sample is only from Guangzhou, one should be cautious when interpreting the representativeness and generalizing the results to other regions. Future investigations are warranted to incorporate data from more regions and healthcare centers. Third, there might be other potential factors (e.g., economic conditions, medical insurance type and coverage, convenience, and discomfort from colonoscopy) that were associated with adherence to colonoscopy but were not available in this study due to the restriction of collected information. Fourth, the questionnaire included all types of intestinal polyps without differentiation, even though only adenomatous polyps are likely to be associated with CRC, which might affect the findings in this study.

In conclusion, our research found that marital status, symptoms of chronic diarrhea and hematochezia, and history of polyps were associated with higher odds of adherence to colonoscopy. On the contrary, histories of chronic diseases were negative factors for adherence to colonoscopy. These findings could help policy‐makers design tailored colorectal cancer screening for these specific populations, and future studies with more representative samples are warranted.

## CONFLICT OF INTEREST

All authors have no conflict of interest to declare.

## AUTHOR CONTRIBUTIONS

Ji‐Bin Li, De‐Sen Wan, Jian‐Hong Peng, and Yu‐Jing Fang conceived and designed the study; Yan‐Ping Wu, Fan Weng, Huan Tian, and Cheng‐Hua Gong acquired the data; Ji‐Bin Li and Keng‐Jian Ke conducted the statistical analyses; Ji‐Bin Li, Wei‐Li Zhang, and Jian‐Hong Peng drafted the manuscript. All authors contributed to the interpretation of the results and critical revision of the manuscript for important intellectual content and approved the final version of the manuscript.

Abbreviations: FIT, fecal immunological test; HRFQ, high‐risk factor questionnaire; SD, standard deviation.

## Supporting information


Table S1‐S2

## Data Availability

The authenticity of this article has been validated by uploading the key raw data onto the Research Data Deposit platform (www.researchdata.org.cn), with the approval RDD number as RDDA2019001156.
