# Supplementary material for: Factors associated with adherence to colonoscopy among individuals who were positive in the preliminary screening for colorectal neoplasms
Source: Cancer Med. 2022 Apr 20;11(22):4321–31. doi: 10.1002/cam4.4730 (PMC9678100; doi:10.1002/cam4.4730)
Supplement: Supplementary file 1 — Table S1‐S2 [file CAM4-11-4321-s001.docx]

Table S1. Factors associated with adherence to colonoscopy among participants positive according to only HRFQ

|  | Adherence to colonoscopy, *n* (%) | Univariate  OR (95%CI) | Multivariable  OR (95%CI) |
| --- | --- | --- | --- |
| Gender |  |  |  |
| Female | 218 (43.0) | 1 | 1 |
| Male | 127 (41.9) | 0.96 (0.72, 1.28) | 0.87 (0.65, 1.18) |
| Age |  |  |  |
| ≤ 60 years | 204 (45.3) | 1 | 1 |
| > 60 years | 141 (39.2) | 0.78 (0.59, 1.03) | 0.81 (0.61, 1.09) |
| Marital status |  |  |  |
| Single/divorce/bereft spouse | 43 (33.9) | **1** | **1** |
| Married | 302 (44.2) | **1.55 (1.04, 2.30)^*^** | **1.55 (1.03, 2.33)^*^** |
| Education level |  |  |  |
| Primary school or below | 21 (28.4) | **1** | ns |
| Middle school | 225 (44.2) | **2.00 (1.17, 3.41)^*^** |  |
| College or above | 99 (43.6) | **1.95 (1.10, 3.45)^*^** |  |
| Smoking |  |  |  |
| Never | 151 (45.8) | 1 | ns |
| Former/current smoker | 28 (45.2) | 0.98 (0.57, 1.68)^†^ |  |
| Alcohol drinking |  |  |  |
| No | 166 (45.2) | 1 | -- |
| Yes | 13 (52.0) | 1.31 (0.58, 2.95) |  |
| History of night work |  |  |  |
| No | 143 (45.7) | 1 | -- |
| Yes | 30 (46.2) | 1.02 (0.60, 1.74) |  |
| Sedentary more than half time in work | |  |  |
| No | 79 (49.1) | 1 | -- |
| Yes | 94 (43.1) | 0.79 (0.52, 1.19) |  |
| History of diabetes |  |  |  |
| No | 157 (46.7) | 1 | -- |
| Yes | 16 (34.8) | 0.71 (0.38, 1.32) |  |
| Overweight or obesity |  |  |  |
| No | 132 (48.2) | 1 | -- |
| Yes | 45 (40.9) | 0.75 (0.48, 1.17) |  |
| **Risk factors** | |  |  |
| First-degree relatives with CRC | |  |  |
| No | 247 (43.6) | 1 | -- |
| Yes | 98 (40.3) | 0.88 (0.65, 1.19) |  |
| Personal history of cancer |  |  |  |
| No | 322 (44.7) | **1** | **1** |
| Yes | 23 (25.8) | **0.43 (0.26, 0.71)^***^** | **0.50 (0.30, 0.84)^**^** |
| History of polypus |  |  |  |
| No | 254 (40.7) | **1** | **1** |
| Yes | 91 (48.9) | **1.40 (1.00, 1.94)^*^** | **1.44 (1.03, 2.02)^*^** |
| Chronic constipation |  |  |  |
| No | 235 (42.0) | 1 | -- |
| Yes | 110 (43.8) | 1.08 (0.80, 1.45) |  |
| Chronic diarrhea |  |  |  |
| No | 237 (40.3) | **1** | ns |
| Yes | 108 (48.7) | **1.40 (1.03, 1.91)^*^** |  |
| Hematochezia |  |  |  |
| No | 226 (39.3) | **1** | **1** |
| Yes | 119 (50.6) | **1.58 (1.17, 2.15)^**^** | **1.52 (1.11, 2.09)^**^** |
| Chronic appendicitis/appendectomy | |  |  |
| No | 293 (42.4) | 1 | -- |
| Yes | 52 (43.7) | 1.05 (0.71, 1.56) |  |
| Chronic cholecystitis/gallbladder resection | |  |  |
| No | 308 (42.9) | 1 | -- |
| Yes | 37 (40.2) | 0.90 (0.58, 1.39) |  |
| Negative life events |  |  |  |
| No | 288 (42.7) | 1 | -- |
| Yes | 57 (42.2) | 0.98 (0.68, 1.43) |  |

--: not applicable; ns: nonsignificant.

BMI: Body mass index; CRC: Colorectal cancer; HRFQ: High-risk factor questionnaire.

OR: odds ratio; 95% CI: 95% confidence interval.

† *p* < 0.10, * *p* < 0.05, ** *p* < 0.01, *** *p* < 0.001

Table S2. Factors associated with adherence to colonoscopy among participants positive according to only FIT

|  | Adherence to colonoscopy, *n* (%) | Univariate  OR (95%CI) | Multivariable  OR (95%CI) |
| --- | --- | --- | --- |
| Gender |  |  |  |
| Female | 104 (55.3) | 1 | 1 |
| Male | 73 (53.3) | 0.92 (0.59, 1.43) | 0.95 (0.60, 1.51) |
| Age |  |  |  |
| ≤ 60 years | 81 (57.0) | 1 | 1 |
| > 60 years | 96 (52.5) | 0.83 (0.54, 1.29) | 0.83 (0.53, 1.30) |
| Marital status |  |  |  |
| Single/Divorce/Bereft spouse | 9 (34.6) | **1** | **1** |
| Married | 168 (56.2) | **2.42 (1.05, 5.61)^*^** | **2.52 (1.08, 5.90)^*^** |
| Education level |  |  |  |
| Primary school or below | 15 (50.0) | 1 | -- |
| Middle school | 128 (53.1) | 1.13 (0.53, 2.42) |  |
| College or above | 34 (63.0) | 1.70 (0.69, 4.20) |  |
| Smoking |  |  |  |
| Never | 56 (47.9) | 1 | -- |
| Ex-/current smoker | 15 (71.4) | 2.72 (0.99, 7.50) |  |
| Alcohol drinking |  |  |  |
| No | 68 (50.8) | 1 | -- |
| Yes | 3 (75.0) | 2.91 (0.30, 28.71) |  |
| History of night work |  |  |  |
| No | 65 (55.1) | 1 | -- |
| Yes | 7 (36.8) | 0.48 (0.18, 1.29) |  |
| Sedentary more than half time in work | |  |  |
| No | 20 (46.5) | 1 | -- |
| Yes | 51 (54.8) | 1.40 (0.68, 2.88) |  |
| History of diabetes |  |  |  |
| No | 63 (53.9) | **1** | **1** |
| Yes | 6 (31.6) | **0.36 (0.14, 0.98)^*^** | **0.35 (0.13, 0.96)^*^** |
| Overweight or obesity |  |  |  |
| No | 49 (53.9) | 1 | -- |
| Yes | 20 (45.5) | 0.71 (0.35, 1.47) |  |
| **Risk factors** |  |  |  |
| First-degree relatives with CRC |  |  |  |
| No | 177 (54.5) | -- | -- |
| Yes | 0 |  |  |
| History of cancer |  |  |  |
| No | 177 (54.5) | -- | -- |
| Yes | 0 |  |  |
| History of polypus |  |  |  |
| No | 177 (54.5) | **--** | -- |
| Yes | 0 |  |  |
| Chronic constipation |  |  |  |
| No | 163 (55.3) | 1 | -- |
| Yes | 14 (46.7) | 0.79 (0.33, 1.51) |  |
| Chronic diarrhea |  |  |  |
| No | 164 (53.6) | 1 | -- |
| Yes | 13 (68.4) | 1.88 (0.70, 5.06) |  |
| Hematochezia |  |  |  |
| No | 171 (55.3) | 1 | -- |
| Yes | 6 (37.5) | 0.48 (0.17, 1.37) |  |
| Chronic appendicitis/appendectomy | |  |  |
| No | 170 (54.0) | 1 | -- |
| Yes | 7 (70.0) | 1.9 (0.51, 7.83) |  |
| Chronic cholecystitis/Gallbladder resection | |  |  |
| No | 174 (54.6) | 1 | -- |
| Yes | 3 (50.0) | 0.83 (0.17, 4.19) |  |
| Negative life events |  |  |  |
| No | 176 (54.7) | 1 | -- |
| Yes | 1 (50.0) | 0.42 (0.04, 4.62) |  |

--: not applicable.

BMI: Body mass index; CRC: Colorectal cancer; FIT: Fecal immunological test.

OR: odds ratio; 95% CI: 95% confidence interval.

† *p* < 0.10, * *p* < 0.05.
